# SSCI: Self-Supervised Deep Learning Improves Network Structure for Cancer Driver Gene Identification

**DOI:** 10.3390/ijms251910351

**Published:** 2024-09-26

**Authors:** Jialuo Xu, Jun Hao, Xingyu Liao, Xuequn Shang, Xingyi Li

**Affiliations:** 1 School of Computer Science, Northwestern Polytechnical University, Xi’an 710072, China; jialuoxu@mail.nwpu.edu.cn (J.X.); haojun@mail.nwpu.edu.cn (J.H.); liaoxingyu@nwpu.edu.cn (X.L.); shang@nwpu.edu.cn (X.S.); 2 Research & Development Institute of Northwestern Polytechnical University in Shenzhen, Shenzhen 518063, China

**Keywords:** cancer driver genes, self-supervised deep learning, graph learning, network structure enhancement

## Abstract

The pathogenesis of cancer is complex, involving abnormalities in some genes in organisms. Accurately identifying cancer genes is crucial for the early detection of cancer and personalized treatment, among other applications. Recent studies have used graph deep learning methods to identify cancer driver genes based on biological networks. However, incompleteness and the noise of the networks will weaken the performance of models. To address this, we propose a cancer driver gene identification method based on self-supervision for graph convolutional networks, which can efficiently enhance the structure of the network and further improve predictive accuracy. The reliability of SSCI is verified by the area under the receiver operating characteristic curves (AUROC), the area under the precision-recall curves (AUPRC), and the F1 score, with respective values of 0.966, 0.964, and 0.913. The results show that our method can identify cancer driver genes with strong discriminative power and biological interpretability.

## 1. Introduction

In biomedical research, the pathogenesis of cancer is complicated and often attributed to the accumulation of genetic mutations [1,2,3]. Cancer genomics aim to elucidate the relationship between tumors and key genes that drive the initiation and progression of cancer [1,4,5,6]. Therefore, identifying cancer driver genes is crucial for understanding the molecular mechanisms of cancer and advancing precision medicine.

In recent years, numerous computational approaches for identifying cancer driver genes have emerged. For instance, approaches based on frequency like MuSic [7], MutSigCV [8], and OncodriveCLUST [9], typically presuppose that mutations in driver genes exhibit higher recurrence rates across different samples than those observed in non-driver genes, leading to identifying prominently mutated genes as cancer driver genes. Meanwhile, approaches based on networks operate on the premise that rather than being caused by a single genetic mutation, cancer emerges from changes in multiple genes that interact closely and together impact critical biological pathways. Therefore, approaches based on networks identify mutated genes with key roles in biological networks as driver genes by applying network propagation approaches. For example, pgWalk [10], RWRH [11], and BiRW [12] use the network propagation strategy to evaluate the degree of association of gene nodes with diseases. NIDM [13] integrates different types of biological networks and analyzes the dynamic responses of nodes to impulsive signals targeted at specific nodes to identify disease-related genes. While these approaches have proven effective in analyzing the influence of gene mutations, they still present some constraints. More specifically, approaches based on frequency may struggle to identify seldom mutated driver genes because they lack a dependable background mutation frequency. The performance of the approaches based on networks can be degraded by unreliable interactions within biological networks and the exclusion of omics data.

Given the rapid advancements in machine learning (ML), numerous related approaches have achieved remarkable success in identifying cancer driver genes. Fundamentally, ML-based approaches emphasize extracting low-dimensional gene representations from various biological features to identify cancer driver genes. Take TUSON and LOTUS as examples. TUSON [14] employs a LASSO regression model, while LOTUS [15] uses a support vector machine (SVM) to detect pan-cancer driver genes. Additionally, other approaches have been developed to identify specific cancer driver genes, like sysSVM [16] and sysSVM2 [17]. Nonetheless, the majority of current ML-based approaches utilize only omics data to develop gene representations for identifying cancer driver genes, often overlooking the valuable structural information provided by biological networks. Graph neural networks (GNNs), a class of deep learning techniques, perform inference on graph-structured data by integrating the network structure with node features to learn node representations. Their exceptional ability to handle high-dimensional and complex biological data makes GNNs particularly well-suited for identifying cancer driver genes.

Some current works have attempted to integrate multi-omics data and biological networks to mine more information using GNNs. EMOGI [18] pioneers the employment of a graph convolutional network (GCN) [19] to learn the representation of gene nodes from protein–protein interaction (PPI) networks and multi-omics data. MTGCN [20] integrates biological features and structural features to create enhanced features for each gene; it employs a multi-task learning framework based on ChebNet [21], aiming to optimize both the primary task and the auxiliary task. SMG [22] employs a pretrain-finetune paradigm. During the pre-training phase, SMG uses the EMOGI strategy to construct multi-omics-featured PPI networks and then randomly masks some nodes. A GNN-based encoder is subsequently used to reconstruct the masked nodes by leveraging neighborhood information. In the task-specific fine-tuning phase, SMG utilizes the pretrained encoder to embed the PPI networks and applies a task-specific layer to make predictions. Additionally, notable advancements have been made in graph-based approaches, particularly within the realms of computational healthcare and general detection [23,24,25,26]. However, the incompleteness and noise present in PPI networks can significantly impair the performance of these models. Incomplete PPI networks may lack crucial interactions, leading to an incomplete representation of the biological context, while noise can obscure relevant signals and make errors in predictions. These issues can reduce the performance of the models, limiting their effectiveness.

This study presents an advanced method utilizing self-supervised deep learning to improve network structure for cancer driver gene identification (SSCI). Firstly, we employ a positive unlabeled (PU) learning algorithm to infer reliable negative samples. After parameterizing the PPI network, we then employ GCN for node classification. Meanwhile, feature masking is applied to the parameterized network, and GCN is applied to perform feature denoising. Finally, by combining the denoised features with the outcomes of node classification, the PPI network structure is updated for subsequent iterations. The experimental results show that SSCI consistently outperforms state-of-the-art methods in terms of AUROC, AUPRC, and the F1 score. Moreover, further experiments demonstrate that SSCI possesses strong biological interpretability.

## 2. Results

### 2.1. Computational Complexity

Our model integrates a self-supervised learning module into the GCN framework. This self-supervised learning module boasts a linear time complexity, ensuring that it does not impose significant additional computational costs. As demonstrated in Table 1, this design leads to a better performance of our model compared to standard GCN and other baseline models, thereby validating the beneficial effect of incorporating the self-supervised learning module.

### 2.2. Improved Predictive Performance of SSCI

We evaluate the performance of SSCI using AUPRC, AUROC, and the F1 score. To ensure an unbiased evaluation, we perform five-fold cross-validation 10 times across all experiments. For comparison, six baseline models are chosen (GCN [19], GAT [27], Chebnet [21], EMOGI [18], MTGCN [20], and SMG [22]), and all methods are provided with identical input data.

- GCN [19] is the typical graph neural network that processes graph-structured data by aggregating features from a node’s direct neighbors along with its own features. This aggregation captures the local graph structure, allowing the network to learn node representations enriched with neighborhood information.
- GAT [27] is a method based on GCN that utilizes an attention mechanism to extract node features. The attention mechanism in GAT computes a weight for each neighbor based on the feature vector of the node itself and the feature vectors of the neighbors, which allows GAT to assign different weights to the features of neighboring nodes, resulting in a more nuanced representation that reflects the relative importance of the contribution from each neighbor.
- Chebnet [21] is a variant of GCN that utilizes Chebyshev filters. Chebyshev filters offer the advantage of efficiently capturing the most significant frequencies in graph-structured data, which is particularly useful for modeling large-scale and high-order neighborhood information. Compared to the standard GCN, ChebNet enables a more flexible aggregation of information across broader graph neighborhoods.
- EMOGI [18] leverages pan-cancer multi-omics data and the PPI network to derive more useful gene representations. Consequently, it can identify pan-cancer driver genes more accurately.
- MTGCN [20] integrates biological features and structural features to construct enhanced features for each gene, and it proposes a multi-task learning framework, focusing on optimizing the main task of node prediction and the auxiliary task of link prediction. Meanwhile, the framework incorporates a weight learner to automatically balance the contributions of both tasks. To bolster the model’s generalization and robustness, MTGCN randomly omits a few edges while training.
- SMG [22] addresses the scarcity of labeled data for cancer driver gene identification. It adopts the strategy of EMOGI to construct multi-omics-featured PPI networks and then randomly masks some nodes. Finally, it utilizes a GNN-based autoencoder to reconstruct the masked nodes by referring to the neighborhood information. In this way, SMG effectively captures the complex interaction relationships between nodes while preserving topological information. In the task-specific fine-tuning stage, SMG leverages the pretrained GNN encoder to embed PPI networks into the feature graphs and adopts a task-specific layer to make the prediction.

In addition, to evaluate the effectiveness of the self-supervised task, we compare it with two-stage training of SSCI (SSCI-2S) and alternating training of SSCI (SSCI-AN). In both variants, $\theta_{\hat{A}}$ only receives gradients from $GCN_{R}$. For SSCI-2S, $GCN_{R}$ is first trained for 100 epochs to minimize $L_{R}$. Subsequently, $GCN_{C}$ is trained for 1000 epochs with fixed $\theta_{\hat{A}}$. For SSCI-AN, after every five epochs of $GCN_{R}$’s training, $GCN_{C}$ is trained for one epoch; this training process is iterated 1000 times to complete.

From Table 1, it can be observed that SSCI outperforms the compared methods in all evaluation metrics, which demonstrates that SSCI can more precisely identify cancer driver genes.

### 2.3. Robustness Evaluation

In this study, SSCI enhances the PPI network structure based on self-supervised deep learning, which can alleviate the incompleteness and noise of the network. To further evaluate the robustness of SSCI, we investigate the performance of SSCI and other compared methods when edges are randomly removed from the PPI network by 20%, 40%, 60%, 80%, and 100%, respectively.

**Figure 1:**
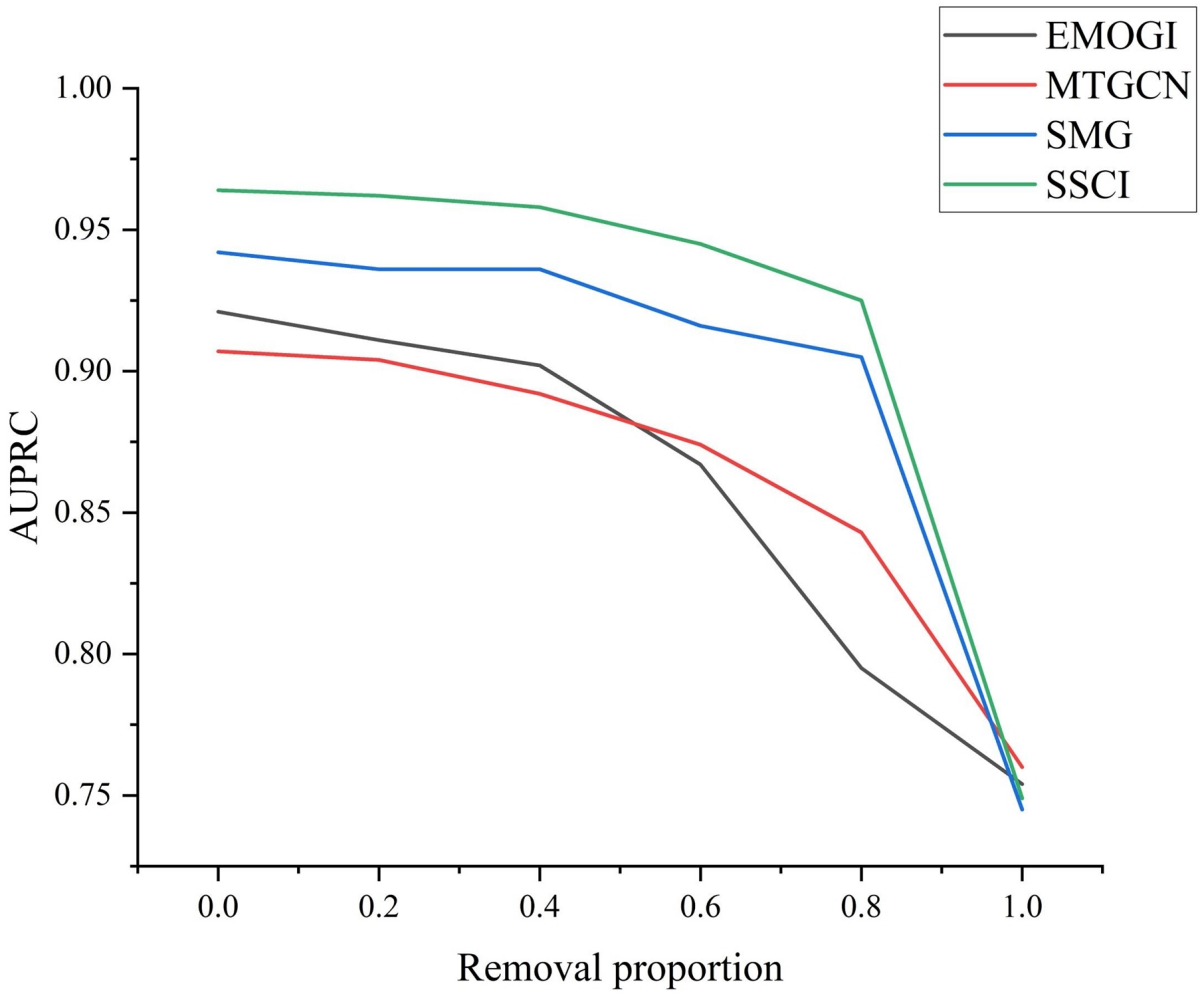
The robustness evaluation of SSCI and the compared methods.

As shown in Figure 1, the results illustrate that SSCI consistently outperforms other methods when they are applied to any processed PPI network, with the exception of the edges dropped out by 100%. A network completely lacking topological information will render the self-supervised task ineffective, so the performance of SSCI is not remarkable when the removal proportion is 100%.

### 2.4. Analysis of Potential Cancer Driver Genes

**Figure 2:**
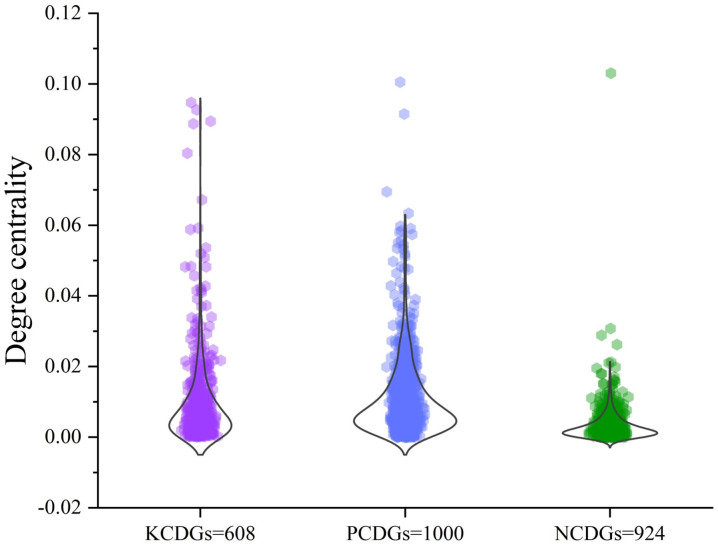
Comparison of the degree centrality of SSCI-PCDGs with KCDGs and NCDGs.

To compare the topological similarity of the predicted cancer driver genes (PCDGs) with that of known cancer driver genes (KCDGs), we further compute centrality scores for nodes within each network and derive the average centrality score for each node. As shown in Figure 2, the degree centrality scores of KCDGs notably exceed those of non-cancer driver genes (NCDGs), implying the importance of these KCDGs within biological networks. Meanwhile, the degree centrality distribution of PCDGs and KCDGs show a high degree of similarity, indicating that cancer driver genes predicted by SSCI share similar topological characteristics with KCDGs but have significant differences in topological structure compared to NCDGs.

### 2.5. Enrichment Analysis

**Figure 3:**
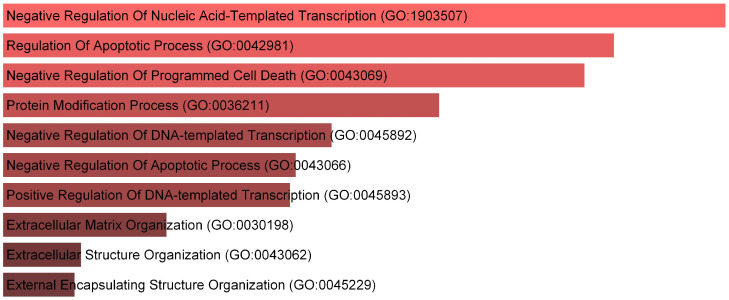
The top 10 biological processes with the highest enrichment.

We also analyze gene ontology (GO) [28] enrichment of the top 100 PCDGs in biological processes (BP) using Enrichr [29,30] (https://maayanlab.cloud/Enrichr, accessed on 14 September 2024). As shown in Figure 3, genes can be significantly enriched in some biological processes which are associated with cancer. For instance, the p53 transcription factor can influence the acute anti-cancer effects of telomerase inhibitors, such as MST-312 [31]. Furthermore, the dysregulation of apoptosis is a critical factor in the development of cancer [32,33,34].

### 2.6. Drug Sensitivity Analysis

**Figure 4:**
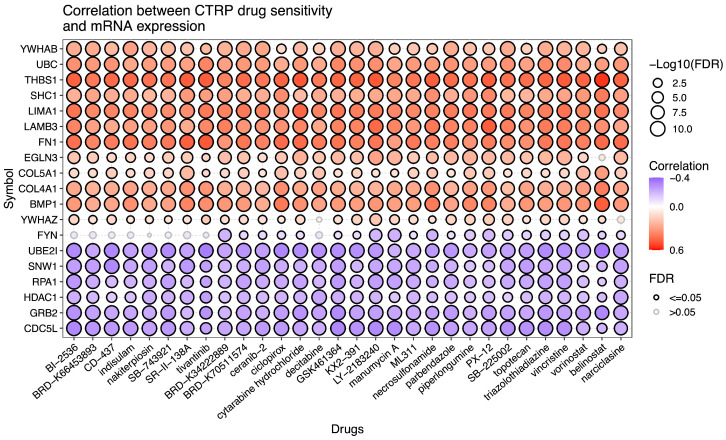
The correlation between gene expression and the sensitivity of drugs (top 30).

We also select the top 20 PCDGs for the Cancer Therapeutics Response Portal (CPTR) [35,36,37] drug sensitivity analysis using Gene Set Cancer Analysis (GSCA, http://bioinfo.life.hust.edu.cn/GSCA, accessed on 14 September 2024) [38,39]. We demonstrate that cancer driver genes identified by our method can provide insight into potential drug targets and contribute to the enhanced efficacy and specificity of cancer therapy. As shown in Figure 4, most genes significantly correlate with drug sensitivity, indicating their potential roles in modulating the response to specific cancer drugs. For example, BI 2536 demonstrates significant efficacy in inhibiting the growth of human tumor xenografts in nude mice and promoting the regression of large tumors when administered through well-tolerated intravenous dosing protocols [40]. CD-437 effectively induces S-phase cell cycle arrest and apoptosis in both androgen-dependent and androgen-independent human prostate cancer cell lines [41]. PX-12 inhibits the growth of A549 lung cancer cells by inducing G2/M phase arrest and promoting Bax-mediated, ROS-dependent apoptosis [42].

## 3. Discussion

Cancer is a significant threats to human health today, with its mechanisms of onset being highly complex. In the field of biomedicine, a widely accepted theory posits that cancer arises from the accumulation of mutations in multiple genes. Consequently, identifying cancer driver genes is crucial for revealing the mechanisms underlying cancer development.

In this paper, we present an advanced approach named SSCI for identifying cancer driver genes using self-supervision in GCN. This approach effectively strengthens the network structure and enhances both predictive accuracy and model robustness. SSCI will aid researchers in understanding the biological characteristics of tumors and provide a more comprehensive insight into the complex processes of cancer progression.

Building on this foundation, we will continue to explore how to more precisely identify cancer driver genes. Our attention will shift to single-cell resolution data. Single-cell data can reveal the role of cancer driver genes from multiple perspectives, including transcriptional regulation, chromosome structure and interactions, as well as cellular heterogeneity. Therefore, by integrating Hi-C data, histone modification data, such as H3K4me3 and H3K27ac, and ATAC-seq data, we hope to gain deeper insights into the mechanisms underlying cancer initiation and progression.

## 4. Materials and Methods

### 4.1. Data Collection

We collect multi-omics data, including gene expression data, DNA methylation data, and single nucleotide variation (SNV) data from The Cancer Genome Atlas (TCGA, https://portal.gdc.cancer.gov/, accessed on 22 June 2024). Our study focuses on 16 types of cancer, including bladder urothelial carcinoma (BLCA), breast invasive carcinoma (BRCA), cholangiocarcinoma (CHOL), colon adenocarcinoma (COAD), esophageal carcinoma (ESCA), head and neck squamous cell carcinoma (HNSC), kidney renal clear cell carcinoma (KIRC), kidney renal papillary cell carcinoma (KIRP), liver hepatocellular carcinoma (LIHC), lung adenocarcinoma (LUAD), lung squamous cell carcinoma (LUSC), pancreatic adenocarcinoma (PAAD), prostate adenocarcinoma (PRAD), rectum adenocarcinoma (READ), thyroid carcinoma (THCA), and uterine corpus endometrial carcinoma (UCEC).

For gene expression data, we download fragments per kilobase of transcript per million fragments of mapped (FPKM) data. Genes with zero expression in more than 10% of the total samples are removed. For DNA methylation data, we download Illumina Human Methylation 450 data and exclude cytosine–phosphate–guanine (CpG) sites exhibiting missing data in over 10% of the total samples. For all data, we exclude formalin-fixed samples, as previous studies have shown that formalin-fixed tissue may affect DNA information compared to fresh frozen tissue [43,44].

By applying the processing procedures described in EMOGI [18], we encode each gene as a 48-dimensional feature vector. For each gene, we calculate the differential expression, differential DNA methylation, and SNV frequency in a specific type of cancer, resulting in a three-dimensional feature vector. By concatenating these vectors across all cancer types, we obtain a 48-dimensional gene feature vector. Finally, we apply z-score normalization to all feature dimensions for each gene.

The differential expression value is calculated by the log2-fold change between the expression of the tumor and normal samples from the same patient:

(1) $$de_{i}^{c}=\frac{1}{N_{de}}\sum_{p\in P_{c}}log_{2}\left(\frac{V_{pi}^{t}}{V_{pi}^{n}}\right),$$

where $V_{pi}^{t}$ and $V_{pi}^{n}$ are the gene expression values for the tumor and the normal sample, respectively, from the patient *p* for gene *i* in cancer type *c*. $N_{de}$ is the number of patients who have both tumor and normal samples in the gene expression data. $P_{c}$ is the set of patients with cancer type *c*.

Differential DNA methylation refers to the disparity in methylation beta values between tumor and normal samples derived from the same patient, which can be quantified as follows:

(2) $$dm_{i}^{c}=\frac{1}{N_{dm}}\sum_{p\in P_{c}}\left(\beta_{pi}^{t}-\beta_{pi}^{n}\right),$$

where $\beta_{pi}^{t}$ and $\beta_{pi}^{n}$ are the DNA methylation values for the tumor sample and the normal sample, respectively, from the patient *p* for gene *i* in cancer type *c*. $N_{dm}$ is the number of patients who have both tumor and normal samples in the DNA methylation data.

SNV frequency refers to the count of non-silent SNVs within a gene, which can be calculated as follows:

(3) $$sf_{i}^{c}=\frac{1}{|P_{c}|}\sum_{p\in P_{c}}F_{p,i},$$

where $F_{p,i}$ is the mutation frequency for the sample from the patient *p* for gene *i* in cancer type *c*. $|P_{c}|$ is the size of the patient set for cancer type *c*.

The PPI network is collected from ConsensusPathDB (CPDB, http://consensuspathdb.org, accessed on 14 September 2024) [45]. Edges with a confidence level higher than 0.5 are preserved. After processing, the network contains 9852 nodes and 336810 edges.

In this study, KCDGs regarded as positive samples are collected from the Network of Cancer Genes (NCG, http://www.network-cancer-genes.org/, accessed on 14 September 2024) [46], the Catalogue of Somatic Mutations in Cancer (COSMIC, https://cancer.sanger.ac.uk/cosmic, accessed on 14 September 2024) [47], and DigSee (http://digsee-digchem.org/geneSearch/, accessed on 14 September 2024) [48].

### 4.2. Negative Sample Inference

To obtain NCDGs, we first employ DeepWalk [49] to extract the topological features of each gene in the PPI network. Subsequently, we employ the PU learning algorithm to deduce credible negative samples, which have a minimal association with cancer. DeepForest [50] is used as the classifier, which learns from the gene topological features to classify KCDGs and assumed NCDGs. During the prediction phase, DeepForest predicts the non-training samples, ranks the prediction results, and then considers genes with high confidence scores as reliable NCDGs. We randomly select five assumed non-cancer driver gene sets from unknown samples and obtain 924 negative samples in total after five iterations. The detailed procedure of PU learning is shown in Algorithm 1.
**Algorithm 1** Positive unlabeled learning algorithm for negative sample inference 1:**procedure** Positive unlabeled learning(P, U) ▹ P is the set of positive samples, U is the set of unknown samples. 2:     Set the maximum number of iterations $i_{max}$.                        ▹ We set $i_{max}$ to 5. 3:     Create empty RN.                      ▹ RN is the set of reliable negative samples. 4:     i=1 5:     **while** $i\leq i_{max}$ **do** 6:          Initialize a classifier. 7:          Randomly select 20% of samples from U as AN.        ▹ AN is the set of assumed negative samples. 8:          Train the classifier using P and AN. 9:          Predict the remaining genes using the classifier. Select the 10% genes with the highest predicted negative samples ranking and add them to the RN.10:          i=i+111:    **end while**12:    **return** RN13:**end procedure**

### 4.3. Network Structure Improvement

**Figure 5:**
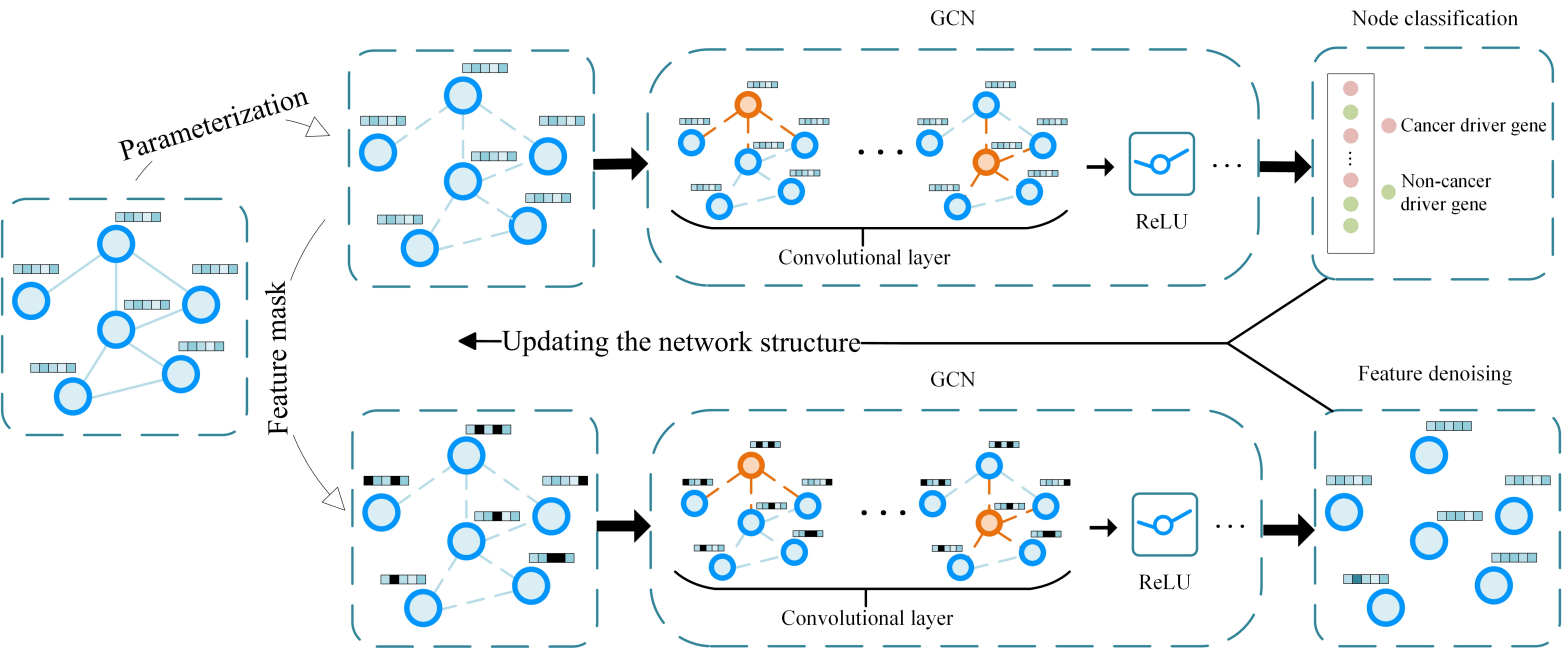
An overview of SSCI (The orange nodes in the figure represent the aggregation of information from their neighbor nodes). The initial step involves parameterizing the PPI network and employing GCN for node classification. Meanwhile, feature masking operations are applied to the parameterized network, and GCN is also utilized to perform feature denoising, thereby enhancing the model’s robustness against noise. The denoised features are then combined with the outcomes of node classification to iteratively update the PPI network structure. Through this iterative process, the structure of the network is continuously refined, optimizing the model’s performance and improving its ability to identify cancer driver genes.

The incompleteness and noise issues in PPI networks weaken the identification effect of cancer driver genes. Inspired by SLAPS [51], we introduce self-supervised learning to improve the structure of the PPI network based on denoising autoencoders [52], and the overview of SSCI is shown in Figure 5.

The PPI network is denoted as an undirected graph G={V,A,X}, where *V* is the set of nodes in the network, and |V|=n represents the number of nodes. $A\in R^{n\times n}$ is the adjacent matrix of the PPI network. $X\in R^{n\times m}$ is the feature matrix of n nodes. $\hat{A}$ is the row-and-column normalized adjacency with self-loops, which can be calculated as follows:

(4) $$\hat{A}=D^{-\frac{1}{2}}\left(A+I\right)D^{-\frac{1}{2}},$$

where *I* is an identity matrix, and *D* is the degree matrix of A+I.

Employing GCN to extract topological information from nodes within a deficient PPI network will weaken performance. Here, we parameterize the PPI network so that it can be dynamically adjusted based on gradients. We initialize each element of $\theta_{\hat{A}}\in R^{n\times n}$ with the corresponding element from $\hat{A}$. During the propagation phase in GCN, $\theta_{\hat{A}}$ is input into each layer of GCN to ensure the network structure can be adjusted based on gradients. The propagation rule between the layers can be defined as follows:

(5) $$H^{\left(l\right)}=\sigma \left(\theta_{\hat{A}}H^{\left(l-1\right)}W^{\left(l\right)}\right),$$

where σ is an activation function such as ReLU [53], $W^{\left(l\right)}$ is the weight matrix in layer *l*, and $H^{\left(l-1\right)}$ is the node representations in layer *l*. $H^{\left(0\right)}$ is the input of the first layer, and $H^{\left(0\right)}=X$.

We use a three-layer GCN as the function $GCN_{C}:R^{n\times m}\times R^{n\times n}\to R^{n\times |C|}$ to identify cancer driver genes. In this study, |C| is equal to 2, owing to the presence of two gene categories: cancer driver genes and NCDGs. The cross entropy loss is employed for node classification, which can be calculated as follows:

(6) $$L_{C}=-\left(ylog\left(\hat{y}\right)+\left(1-y\right)log\left(1-\hat{y}\right)\right),$$

where $\hat{y}$ is the predicted probability of the node from the output of the $GCN_{C}$, and *y* is the true label of the node (cancer driver genes or NCDGs).

The PPI network is sparse, consisting of 9852 nodes and 336810 edges. In this case, labeled nodes cannot effectively supervise all nodes in the PPI network. In addition, the sparsity of the network partly increases the impact of noise in the network, which weakens the performance. Self-supervised deep learning can reduce dependence on labeled data and capture more detailed information from data. In this study, we perform a self-supervised task based on denoising autoencoders. At first, we randomly mask some node features, and masked features can be calculated as follows:

(7) $$X^{\prime }=M\cdot X,$$

where $X^{\prime }$ is the masked feature. $M\in R^{n\times m}$ is the matrix of masked indices, $M_{i,j}$ independently obeys $M_{i,j}∼Bernoulli\left(1-\gamma \right)$, and γ is the percentage of the mask indices. We use a three-layer GCN as the function $GCN_{R}:R^{n\times m}\times R^{n\times n}\to R^{n\times m}$ to recover origin node features. In this case, the mean square error is employed, which can be calculated as follows:

(8) $$L_{R}=\frac{1}{\sum_{i=1}^{n}\sum_{j=1}^{m}M_{ij}}\sum_{i=1}^{n}\sum_{j=1}^{m}\left[M_{ij}{\left(X_{ij}-{\hat{X}}_{ij}\right)}^{2}\right],$$

where $\hat{X}\in R^{n\times m}$ are the denoised features from the output of $GCN_{R}$.

Our model is trained to minimize *L*, which can be calculated as follows:

(9) $$L=L_{C}+\lambda L_{R},$$

where λ is a hyperparameter used to balance two tasks.

### 4.4. Hyperparameter Setting

In this study, the labelled data are randomly split into a training set (80%), validation set (10%), and test set (10%). The validation set is used for hyperparameter optimization and model selection. We choose Adam [54] as the optimizer for SSCI and other GNN-based baselines. The best hyperparameter combination for SSCI is as follows: the $GCN_{C}$ has three convolution layers, including 128, 128, and 2 neurons, respectively, the $GCN_{R}$ has three convolution layers, including 128, 128, and 48 neurons, respectively, the feature dropout rate and edge dropout rate are both 0.5, the learning rate of $GCN_{C}$, $GCN_{R}$, and $\theta_{\hat{A}}$ are 0.001, 0.001, and $10^{-7}$, the weight decay of $GCN_{C}$, $GCN_{R}$, and $\theta_{\hat{A}}$ are 0.002, 0, and 0, λ is 0.1, γ is 0.05, and the number of training epochs is 1000. GNN-based baselines have three layers, including 128, 128, and 2 neurons, and the training epoch, feature dropout rate, learning rate, and weight decay are 1000, 0.5, 0.001, and 0.002, respectively. The parameters of comparison methods are configured in accordance with the guidelines provided in their respective publications.

## 5. Conclusions

The identification of cancer driver genes is of paramount importance for unraveling the complex biological mechanisms of the development, progression, and response to therapy in cancer. The availability of extensive omics data and interactome networks from various comprehensive databases has facilitated the deployment of graph deep learning techniques. Nonetheless, the majority of existing models inadvertently overlook the inherent incompleteness and noise within the biological networks; such oversights may significantly affect the accuracy and reliability of the model.

In this study, we proposed a novel method to integrate self-supervised learning with the cancer driver gene identification process, which effectively refined the network architecture, leading to a significant enhancement in model performance and robustness. SSCI demonstrated its excellence and robustness in predictive tasks, outperforming other baseline models, as evidenced by the results. Moreover, topological similarity analysis, enrichment analysis, and drug sensitivity analysis substantiated that the majority of genes identified by SSCI are indeed related to the occurrence and progression of cancers. In addition, we posit that the versatility of SSCI extends beyond cancer analysis, offering a generalizable approach for the study of a spectrum of complex diseases. The source code is available on GitHub at https://github.com/xingyili/SSCI. 

## Figures and Tables

**Table 1:** Predictive performance of SSCI compared to other methods for cancer driver gene identification.

METHOD	AUPRC	AUROC	F1 Score
GCN	0.921	0.947	0.877
GAT	0.938	0.956	0.887
Chebnet	0.940	0.945	0.866
EMOGI	0.921	0.946	0.876
MTGCN	0.907	0.921	0.822
SMG	0.942	0.951	0.876
SSCI-AN	0.955	0.958	0.891
SSCI-2S	0.956	0.958	0.891
SSCI	0.964	0.966	0.913

## Data Availability

The datasets and materials used during the current study are available from the corresponding author upon reasonable request.
